# ‘Getting back to normality seems as big of a step as going into lockdown’: the impact of the COVID-19 pandemic on people with early to middle stage dementia

**DOI:** 10.1093/ageing/afab012

**Published:** 2021-01-22

**Authors:** Catherine V Talbot, Pam Briggs

**Affiliations:** Department of Psychology, Northumbria University, Newcastle upon Tyne, UK; Department of Psychology, Bournemouth University, Poole, UK; Department of Psychology, Northumbria University, Newcastle upon Tyne, UK

**Keywords:** Alzheimer’s, cognitive impairment, outdoors, social inclusion, older people, Covid-19, qualitative

## Abstract

People with dementia can experience shrinkage of their social worlds, leading to a loss of independence, control and reduced well-being. We used ‘the shrinking world’ theory to examine how the COVID 19 pandemic has impacted the lives of people with early to middle stage dementia and what longer-term impacts may result. Interviews were conducted with 19 people with dementia and a thematic analysis generated five themes: the forgotten person with dementia, confusion over government guidance, deterioration of cognitive function, loss of meaning and social isolation, safety of the lockdown bubble. The findings suggest that the pandemic has accelerated the ‘shrinking world’ effect and created tension in how people with dementia perceive the outside world. Participants felt safe and secure in lockdown but also missed the social interaction, cognitive stimulation and meaningful activities that took place outdoors. As time in lockdown continued, these individuals experienced a loss of confidence and were anxious about their ability to re-engage in the everyday practises that allow them to participate in society. We recommend ways in which the government, communities and organisations might counteract some of the harms posed by this shrinking world.

## Key Points

- The COVID-19 pandemic has shrunk the social worlds of some people with dementia.
- Some people with dementia felt safe and secure in lockdown as it provided a break from the stresses of everyday life.
- As time in lockdown continued, people with dementia felt they were losing the ability to participate in society.
- People with dementia experienced a loss of social interaction, cognitive stimulation and purpose during lockdown.
- There appeared to be a lack of support for helping people with dementia re-enter the world after lockdown.

## Introduction

The COVID-19 pandemic has caused significant global disruption. People with dementia may be vulnerable to the impacts of the pandemic and associated lockdown due to a disruption of routines, lack of cognitive stimulation, reduced social connection, and increased levels of anxiety and depression [1, 2]. Indeed, Giebel *et al*. [3] found that carers and people with dementia reported faster deterioration, which they attributed to a lack of stimulation and social contact. Research conducted with people with dementia can help us to understand the impact that the pandemic has had on their lives. This could then inform the development of services, healthcare, legislature, and interventions to support this population in the event of future pandemics.

People with dementia may also experience challenges when transitioning out of lockdown after being confined to their homes for a substantial period of time [2]. Duggan *et al*.’s [4] shrinking world theory appears to be directly relevant to the pandemic, where the shrinking world refers to a reduction in the number of places a person with dementia feels comfortable. In turn, this can lead to an overall decline in independence, control and well-being. Other researchers have explored how people with dementia perceive outside spaces, identifying tensions between the outdoors being both therapeutic and a source of anxiety [5]. Anxiety about the outdoors can result in a loss of confidence and social activities being halted, which is problematic given that interactions outside are an important source of identity, social inclusion and well-being [4,5]. The pandemic may exacerbate the shrinking world effect and pose challenges for people with dementia once their worlds can ‘expand’ again post-lockdown.

**Table 1:** Participant information

ID	Gender	Age	Living situation	Type of dementia
P1	Female	58	Living with family	Alzheimer’s disease
P2	Male	59	Living with family	Mixed
P3	Male	84	Living with family	Alzheimer’s disease
P4	Female	55	Living with family	Alzheimer’s disease
P5	Male	57	Living with family	Mixed
P6	Male	61	Living with family	Vascular
P7	Female	62	Living with family	Alzheimer’s disease
P8	Male	62	Living with family	PCA^a^
P9	Male	63	Living with family	Frontotemporal
P10	Male	62	Living independently	PCA^a^
P11	Male	68	Living independently	Alzheimer’s disease
P12	Male	55	Living with family	Alzheimer’s disease
P13	Female	64	Living with family	Vascular
P14	Male	62	Living with family	Vascular
P15	Male	68	Living with family	Vascular
P16	Male	67	Living with family	Alzheimer’s disease
P17	Female	66	Living independently	Mixed
P18	Female	50	Living independently	PCA^a^
P19	Female	64	Living independently	Mixed

^a^Posterior cortical atrophy.

The pandemic will likely have long-lasting effects on the lives of people with dementia, and the voices of people with dementia will be instrumental in understanding these impacts. We centre the voices of people with dementia in our research and ask: ‘How has the COVID-19 pandemic impacted the lives of people with dementia’.

## Method

### Sample

We aimed to recruit 12–20 participants, consistent with recommendations for interview studies that aim to identify patterns across data [6]. Nineteen participants were recruited via Twitter, Facebook, dementia organisations and networks, peer support and advocacy groups. Inclusion criteria were as follows: self-identify as a person with dementia; live in the UK; be able to demonstrate capacity. Participants were aged between 50 and 84 years (*M* = 62.47 years, *SD* = 7.05 years). Participants were relatively young, with 14 having young-onset dementia (i.e. living with a diagnosis made before the age of 65). Seven identified as female and 12 as male. Fourteen participants lived with family members and five lived independently. Table 1 provides demographic information about the participants.

### Procedure

After consent was obtained and capacity demonstrated, the first author (C.V.T.) conducted semi-structured interviews with participants. Interviews were conducted between June–July 2020, as lockdown restrictions started to ease in the UK. Interviews were conducted remotely due to the pandemic. Participants chose their preferred interview format. Nine interviews were conducted using video-calling software, nine by telephone and one by email.

Interviews were guided by an interview protocol, which included questions on experiences of the pandemic, challenges of the pandemic, coping mechanisms and feelings about transitioning out of lockdown. For the email interview, the participant requested that questions be sent individually. This enabled us to follow-up on points of inquiry and seek clarification, while also providing an accessible way for the participant to take part in the study. Interviews lasted between 30 and 60 min and were recorded using an encrypted digital dictaphone. Interview data were then transcribed verbatim and anonymised.

### Analysis

Transcripts were analysed thematically, following the six steps outlined by Braun and Clarke [7]. C.V.T. began by reading and re-reading interview transcripts while making notes of coding ideas. The data were then imported into NVivo12, and C.V.T. coded the data independently. Interview data were coded deductively and semantically, with codes reflecting the explicit content of the data. Codes were then collated and examined to identify patterns of meaning across the data. Initial themes were then generated and sent to the second author (P.B.) for critical feedback, along with all data aligned to these themes. The authors then worked collaboratively to finalise the themes. These themes were then presented to a group of people with dementia, who provided critical feedback. Amendments were then made where appropriate.

## Themes

Five themes were generated from the data: the forgotten person with dementia; confusion over government guidance; deterioration of cognitive function; loss of meaning and social isolation; safety of the lockdown bubble. We discuss these themes below, using Duggan *et al*.’s [4] shrinking world theory to interpret the data. We outline the direct impacts of the pandemic on people with dementia and the tensions this creates when transitioning out of lockdown, with many feeling that ‘getting back to normality seems as big of a step as going into lockdown’.

### The forgotten person with dementia

Participants reported that society had failed to support people with dementia during the pandemic, with many feeling ‘forgotten’ and ‘abandoned’ by the government, local authorities and NHS. Most felt there was a lack of support for people with dementia and the onus was on them to actively seek out support:

*‘*Nobody has actually contacted me in any way to make sure that I’m still alive. Everyone that I’ve had help from, like the food parcels and things like that, I’ve had to initiate myself’ P15

People with dementia were not included in the UK Government’s priority list of those ‘most vulnerable’ individuals that needed protection and consequently could not access some of the benefits of those who’d been asked to ‘shield’ themselves. They felt forgotten and struggled to understand how they could be ignored:

*‘*I've heard a lot of examples where people who are shielding have been offered help by local authorities, we were helped by nobody, and if we didn't have family to help us, we would be struggling’ P6

One benefit denied to participants was the ability to go shopping during the hours dedicated to those on the priority list. This was a key issue for many participants*,* who did not understand why they were not recognised as needing help for this kind of stressful activity*:*

*‘*I’m only 55 years old so I’m not in the category of being vulnerable even though we are vulnerable. I don’t have any underlying health problems so for me, and I know for many others, they’ve found that the supermarket shopping was horrendous.’ P4

In particular, participants felt dementia-friendly practises had been abandoned by supermarkets, with many finding procedures and signage to be challenging. For example, P4 described an unpleasant experience at a supermarket:

*‘*I went through the doorway, the two members of staff shouted at me which I got a little bit anxious and upset and confused and I didn’t know what I was doing. ‘You shouldn’t be coming out of this door get back in there!’ and I just went, I just ran with my trolley. So, that sort of unnerved me a little from going back. So, hubby does that now’ P4

P4’s experience caused her to stop going to the supermarket, thereby shrinking her world and impacting her independence. It is therefore vital that dementia-friendly practises are not abandoned in the event of future pandemics.

In the context of shrinking worlds, these findings suggest that people with dementia have been forgotten during the pandemic, resulting in a lack of support to help them venture into the world post-lockdown. A lack of support at a societal level could impede a person’s ability to participate in society, thus impacting their independence and contributing to their social exclusion.

### Confusion over government guidance

Many people have found government guidance to be confusing during the pandemic [8]; however, this information seemed to pose greater challenges for participants, who found the guidance completely inaccessible:

‘I don't think the message is clear. I don't think that people with dementia understand it. You have this communication issue anyway. The message is not concise, they're for everybody, they're not catered for people with illnesses’ P9

Keeping up with government guidance was cognitively taxing for participants. For example, P1 said ‘it tired me out because you get conflicting news’. A lack of accessible guidance not only socially excludes people with dementia but could also put them at increased risk of getting COVID-19.

As a result of being unable to understand government guidance, some participants became more reliant upon family members:

*‘*If I was living on my own, I would have no idea what was going on because it was so airy-fairy and common sense here and thank goodness that [carer] was here cause occasionally I have had to say can we do that?’ P13

This suggests that inaccessible guidance not only adds to caregiver responsibilities but also negatively impacts a person’s independence. Participants recognised that clearer government guidance could promote their independence, with many advocating for guidance to be more concise, communicated at a slower pace and shared in alternative dementia-friendly formats:

*‘*We need to keep our independence—should be more clarity—you know when they understand the way dementia is—there should be more clarity and direction in clear and simple terms’ P11

These findings indicate that there are small steps that the UK government could take to improve communication during future pandemics. This would not only be beneficial for people with dementia but also for wider society. A lack of accessible guidance could limit the ability of people with dementia to participate in society post-lockdown, thereby contributing to their shrinking worlds.

### Deterioration of cognitive function

Participants reported a deterioration of cognitive function, describing changes in speech, memory, concentration and balance, which they attributed to a lack of cognitive stimulation:

‘That was the hardest bit, because my memory has become really bad since we stopped doing stuff because I’ve been doing mundane tasks’ P12

Understandably, some participants felt anxious about a perceived loss of skills and wondered whether they would be able to regain former cognitive function:

‘Whether I’ve lost any skills I had before that prevent me from doing the things I did…For me the main challenge is, is the unknown of what I don’t know yet, what I won’t be able to do. If I can relearn then that will be a bonus, but it’s that uncertainty of not knowing’. P19

Participants also attributed a loss of routine to symptom progression, which they tended to discuss in relation to speech. They explained that they were not having as many conversations during the pandemic and felt this had affected their speech:

‘The way I talk has become quite different, I think. Probably because I haven't been talking to as many people which I sort of preferred but probably didn't realise how much good it was doing until I wasn't doing it’ P4

This suggests that a lack of rehearsal and reinforcement may have caused some individuals’ dementia to progress, resulting in a loss of abilities. These self-reported changes were a source of anxiety for participants, which impacted their confidence to participate in society post-lockdown. One key concern related to their ability to travel:

Which is the worry for when we are allowed to travel, you know will I have forgotten how to book a train ticket or travel and things? My brain has definitely slowed down because it’s not been exposed to the stuff that I was before. P19

Not being able to travel is problematic for people with dementia because it immediately shrinks their social world, thereby halting social activities and reducing opportunities for social interaction.

In the context of shrinking worlds, these findings suggest that a lack of rehearsal of everyday practises has caused some individuals to feel like they have lost some of the skills required to venture into the outside world, potentially halting the expansion of their worlds post-lockdown.

### Loss of meaning and social isolation

For many participants, the pandemic led to a loss of self-worth. They reported that a loss of purpose stemmed from being unable to take part in meaningful activities, such as attending walking groups, visiting allotments and engaging in advocacy. These activities were cancelled during the pandemic and, consequently, participants felt unvalued:

‘We talked about why I felt so low and it was about this sense of not being able to contribute anymore’ P13

A loss of purpose was a key concern for individuals who engaged in advocacy, which can provide some people with a way of regaining purpose and respect, and ‘fighting back’ against the condition [9]. Not being able to engage in advocacy work was challenging for participants, resulting in a loss of value, identity and reduced well-being:

*‘*These things were being taken out and my diary was completely empty. Whilst I have a great life, part of that is the advocacy work which gives me a sense of wellbeing. I have had such positive responses, I know it’s doing good, so it felt like my purpose had been taken away’. P13

Participants also reported that a loss of purpose contributed to mental health difficulties, with some describing experiences of depression:

‘Before I had a diary about what I'm doing but the diary went blank. Difficult to replace it…When you get stuck indoors for three months, it started playing on me and depression came up because I was home all the time. I was so down with depression’ P14

Other participants expressed that they felt more socially isolated or lonely as they missed the social interactions that were facilitated by meaningful activities:

‘I think the biggest issue is the loneliness. Although I've got my carer, he's a little years older than me. So that in itself is, even is—not been able to attend any groups’ P7

These findings suggest that the shrinking world effect that seems to have been exacerbated by the pandemic has had a detrimental impact on participants’ sense of identity and social connectedness. It is important that people with dementia are supported when returning to meaningful activities and that these activities are accessible.

### Safety of the lockdown bubble

There have been concerns that increases in social isolation during lockdown periods may lead to chronic loneliness, which could negatively impact mental health and well-being [10,11]. While this was true for participants, they also reported gaining feelings of safety and security in the ‘lockdown bubble’. Although participants recognised that their worlds had shrunk since lockdown, they also felt that this had provided them with a welcome break from the noise and busyness of everyday life, which was often tiring and overloading of their senses:

“It's been okay because it's like our world has shrunk a bit, because for me it’s a good thing because I couldn't handle crowds and lots of conversation” P12

Others found relief in being able to be their true self during lockdown, where they no longer needed to try to act ‘normal’:

‘No, and I forgot how difficult it was to get up and join in and be normal. You know and I don’t want to do that now and it was bloody hard work!’ P2

Lockdown provided others with a safe space to learn new skills or return to past hobbies, such as crafting, gardening and photography. In this environment, participants felt they could challenge themselves without fearing failure:

‘I haven’t had to go out of my way, I haven’t had to stress about things, I haven’t had to fail. Which has been a massive part of my life the last few years, it’s just been brilliant’ P2

In comparison to the safe home environment, the outdoors was a source of anxiety for participants, with some expressing that they felt ‘agoraphobic’. P13 stated ‘I don’t want to go anywhere I feel safe here’. Similarly, other participants expressed concerns about their ability to cope with normality, which included loud environments and interacting with others:

‘I'm a little bit anxious about being around a lot of people, I believe I'm right in saying our pub opens soon. I don't really know if I'm that confident about going down there and seeing people I haven't seen for a long while and being around too many people’ P12

Participants also explained that they were anxious about their ability to follow safety procedures in the new post-lockdown world:

‘People aren’t considering that, the problems that people with dementia might have remembering the rules. So, I think that’s stopping a lot of people going out, it’s certainly stopping me from going on the village bus which is back up and running now. I can’t remember the rules for what I have to do’ P19

These findings indicate that there is a tension between the outside world being a source of anxiety and the home environment being a safe space for people with dementia. This tension appears to contribute to the shrinking worlds of people with dementia, making it more challenging for their worlds to expand post-lockdown.

## Discussion

We found that the COVID-19 pandemic has accelerated the ‘shrinking world’ that often accompanies dementia [4]. Despite this shrinking world, people with dementia experienced both losses and gains. They felt safe and secure in their home environment as it provided a break from the stresses of everyday life and created opportunities for achievement. People with dementia also missed the cognitive stimulation, meaningful activities and social interactions that took place outdoors, yet there was little support to help them venture back out into the world, causing many to feel forgotten by society. As time in lockdown continued, people with dementia also felt they were losing control of everyday processes due to a loss of confidence and perceived symptom progression. These findings highlight a tension in how people with dementia perceived the outside world during the pandemic, supporting findings that the outdoors is both therapeutic and a source of anxiety [5]. People with dementia will need to negotiate this tension when deciding whether to expand their social worlds once lockdown restrictions ease. It is essential that these individuals are supported by carers, services, the government, and wider society when making this transition.

Our findings suggest that some peoples’ cognitive function has deteriorated during the pandemic, supporting prior work [3]. This may be due to a lack of stimulation and rehearsal of everyday practises. Brittain *et al*. [5] found that familiarity with the outdoors was key for enabling people with dementia to go outside. Consistent with this, as a result of being confined to their homes during the lockdown, the outside world now seems unfamiliar to some people with dementia, with some feeling like they have lost some of the skills required to venture outdoors. Cognitive rehabilitation may provide one solution to combat this effect, whereby people with dementia could set achievable goals and develop effective strategies to support their ability to participate in a post-lockdown society.

We observed that a lack of meaningful activity during the pandemic was coupled with a loss of self-worth among people with dementia—something associated with ‘living well’ [12]. Other researchers have found that meaningful activity can preserve dignity and a sense of identity [13]. However, our findings suggest that the pandemic has threatened this sense of identity. Given the longevity of the pandemic, it could be some time until people with dementia can return to meaningful activities. Researchers could therefore consider alternative means of promoting a sense of purpose and identity among people with dementia. Digital solutions may hold some promise, with researchers reporting that everyday technologies such as social media provide a way for people with dementia to engage with communities [14]. These technologies could potentially combat the shrinking world imposed by both the pandemic and the diagnosis itself.

We found that people with dementia felt unsupported by society during the pandemic due to a lack of accessible information, a perceived abandonment of dementia-friendly practises and not being included on priority lists. In recent years, we have made great progress in creating dementia-friendly communities [15]; it is important that these practises are not abandoned and people with dementia are not forgotten. A loss of ability to participate in society can be distressing for people with dementia and heighten feelings of isolation [16]. Other researchers have found that people with dementia report feeling anxious about no longer being able to follow the ‘rules of modern life’ when outdoors [17]. This was also true for participants, who felt unsupported and anxious about how to participate in a post-lockdown society. Thus, a lack of accessible information and dementia-friendly practises upon transitioning out of lockdown may serve to socially exclude people with dementia and limit the possibility of their social worlds expanding once again.

The voices of people with dementia will be vital in developing responses to future pandemics, to uphold their social inclusion and independence. We make the following recommendations based on our data: (i) include people with dementia on priority lists; (ii) stores could work with people with dementia to ensure procedures during pandemics (and beyond) are dementia-friendly; (iii) government guidance should be accessible for people with dementia; (iv) services should keep in regular contact with people with dementia, providing support in a variety of formats; (v) cards containing information about safety procedures could be developed for people with dementia, which could serve as a helpful reminder when outdoors. These responses will be instrumental in counteracting the harms of the shrinking world.

There are some limitations of our research. First, we relied on self-reports of diagnoses and symptoms, rather than seeking confirmation from medical records or using operationalised measures. It is possible that participants may not have been aware of some changes in symptoms. In the future, data from large cohort studies could be analysed to determine how the pandemic has impacted cognition, functional ability, self-esteem and a person’s ability to ‘live well’. It might also be beneficial to include carers’ perspectives. However, it should be noted that researchers have demonstrated that people with dementia can rate their functional ability with reasonable accuracy when compared with informants [18].

Recruiting participants via social media and dementia groups may have biassed our sample towards younger people in the early stages of dementia. Thus, participants are not representative of people in the later stages of the disease, nor do they claim to be. Although it was beyond the scope of our work, findings may differ between people with young-onset and late-onset dementia and the early and late stages of the disease. Future work could tease apart these nuances. Moreover, all participants lived independently or with family members; no participants lived in a care home, which have arguably been hit hardest by the pandemic [19]. Our findings are not therefore reflective of the experiences of people living in care homes, who will likely have unique experiences of the pandemic that will be vital in reforming health and social care.

## Conclusions

In conclusion, our findings suggest that the pandemic has accelerated the ‘shrinking world’ effect and created a tension in how people with dementia perceive the outside world. Participants felt safe and secure in lockdown but also missed the social interaction, cognitive stimulation and meaningful activities that took place outdoors. As time in lockdown continued, these individuals experienced a loss of confidence and felt anxious about their ability to re-engage in the everyday practises that allow them to participate in society. It is essential that these individuals are supported by carers, services, the government and wider society when transitioning out of lockdown. We recommend that the voices of people with dementia are included in the development of responses to public health emergencies.
